# *Staphylococcus epidermidis* recovered from indwelling catheters exhibit enhanced biofilm dispersal and “self-renewal” through downregulation of *agr*

**DOI:** 10.1186/1471-2180-12-102

**Published:** 2012-06-08

**Authors:** Lu Dai, Liang Yang, Chris Parsons, Victoria J Findlay, Søren Molin, Zhiqiang Qin

**Affiliations:** 1 Key Laboratory of Arrhythmias, Ministry of Education, Research Center for Translational Medicine, East Hospital, Tongji University School of Medicine, Shanghai, 200120, China; 2 Departments of Medicine, Louisiana State University Health Sciences Center, Stanley S. Scott Cancer Center, 533 Bolivar St., New Orleans, LA, 70112, USA; 3 Microbiology, Immunology, and Parasitology, Louisiana State University Health Sciences Center, Stanley S. Scott Cancer Center, 533 Bolivar St., New Orleans, LA, 70112, USA; 4 Infection Microbiology Group, Centre for Systems Biology, Technical University of Denmark, DK-2800, Lyngby, Denmark; 5 Departments of Pathology and Laboratory Medicine, Medical University of South Carolina, 86 Jonathan Lucas St., Charleston, SC, 29425, USA

**Keywords:** Staphylococcus epidermidis, Biofilm, Autolysis, Extracellular DNA

## Abstract

**Background:**

In recent years, *Staphylococcus epidermidis* ( *Se*) has become a major nosocomial pathogen and the most common cause of infections of implanted prostheses and other indwelling devices. This is due in part to avid biofilm formation by *Se* on device surfaces. However, it still remains unknown that how the process of *Se* biofilm development is associated with relapsed infection in such patients.

**Results:**

We have identified clinical *Se* isolates displaying enhanced biofilm dispersal and self-renewal relative to reference strain. These isolates also exhibit enhanced initial cell attachment, extracellular DNA release, cell autolysis and thicker microcolonies during biofilm development relative to reference strain. Our genetic analyses suggest that these clinical isolates exhibit significant downregulation of *RNAIII*, the effector molecule of the *agr* quorum sensing system, and upregulation of the autolysin gene *atlE*. Isogenic deletion of the *agr* system in *Se* 1457 confirmed that *agr* negatively regulating *atlE* resulted in enhanced initial cell attachment, extracellular DNA release, cell autolysis and biofilm formation abilities. In contrast, double deletion of *agr* and *atlE* significantly abolished these features.

**Conclusions:**

Collectively, these data reveal the role of *agr* system in long-term biofilm development and pathogenesis during *Se* caused indwelling devices-related relapsed infection.

## Background

In recent years, coagulase-negative *Staphylococcus epidermidis* ( *Se*) has become the leading cause of infections related to indwelling medical devices such as vascular catheters, prosthetic joints and artificial heart valves [1,2]. Pathogenicity of *Se* is attributed to its formation of biofilm on the surface of medical devices, thereby enhancing *Se* resistance to antibiotics and host defenses in this setting [3,4]. In general, *Se* biofilm formation is a two-step process, in which bacteria first adhere to the surface (initial attachment phase) and subsequently form cell–cell aggregates and a multilayered architecture (accumulative phase) [5,6]. One autolysin protein, AtlE, facilitates bacterial attachment to the surface of medical devices and dictates pathogenesis for *Se* biofilm-associated infections in vivo [7,8]. In the accumulative phase, the polysaccharide intercellular adhesin (PIA), a linear poly-Nacetyl-1,6-β-glucosamine (PNAG) encoded by the *icaADBC* locus, is the major pathogenic determinant for intercellular adhesion [9,10]. In addition, we have previously demonstrated that extracellular DNA is generated during *Se* growth through AtlE-mediated lysis of a subpopulation of the bacteria; moreover, this process is required for initial bacterial attachment to surfaces and biofilm development [11]. An important negative regulator of biofilm formation by *Se* and *Staphylococcus aureus* is the accessory gene regulator ( *agr*) quorum sensing system, and *agr* mutation promotes biofilm formation by increasing the capacity of *Se* for initial cell attachment [12-14]. The *agr* system of *Se and S. aureus* consists of 4 genes ( *agrAagrCagrD*, and *agrB*) that are cotranscribed (RNAII) and the gene for the effector molecule of the *agr* system, RNAIII, which also encodes the gene for δ-toxin ( *hld*) [12,15].

Medical device-associated biofilms facilitate recalcitrant or recurrent infections despite use of appropriate antibiotics. However, there are only limited data about the long-term *Se* biofilm development, especially clinical isolates recovered from indwelling medical devices infection. It still remains unknown that how the process of *Se* biofilm development is associated with relapsed infection in such patients. Moreover, the molecular mechanisms causing such repeated infection also needs to be investigated. In the current study, we compared the long-term (~7 days) biofilm development and dispersal between *Se* clinical isolates causing indwelling medical devices infection and reference strain in the flow-chamber systems. We also compared the biofilm-related events (initial attachment, PIA synthesis, extracellular DNA release etc.) and biofilm-associated gene profiles in these clinical isolates and reference strain.

## Methods

### Bacterial strains, growth media and reagents

4 *Se* clinical isolates, referred to as *Se*-1, *Se*-2, *Se*-3 and *Se*-4, were recovered from 4 different patients at the Zhongshan Hospital (Shanghai, China) with indwelling catheter-associated infections as defined by the presence of fever, bacterial growth from peripheral blood samples collected from catheter sites. *Se* biofilm-positive strain 1457 wild type and *agr* mutants were kindly provided by Dr. Min Li (Huashan Hospital, Shanghai, China), as described previously [13]. The *agr*/ *atlE* double mutant was constructed as described previously [11]. The mutation was confirmed by Southern blotting and direct sequencing (data not shown), and we also independently confirmed that the 1457 *agr* mutant or *agr*/ *atlE* double mutant does not affect bacterial growth (see Additional file 1: Figure S1). *Se* biofilm-positive ATCC 35984 (also referred as RP62A) and biofilm-negative ATCC 12228 reference strains were purchased from American Type Culture Collection (ATCC). Tryptic soy broth (TSB; Oxoid) medium containing 0.25% glucose was used to support biofilm formation in the microtitre plates. AB medium [16] supplemented with 0.3 mM glucose and 3% TSB was used for biofilm cultivation in the flow-chamber system. SYTO 9 and propidium iodide (PI) (Live_Dead reagents, Molecular Probes) were used at a concentration of 1 μM for staining live or dead bacteria in biofilms, respectively. DDAO [7-hydroxy-9 H-(1,3-dichloro-9,9-dimethylacridin-2-one)] (Molecular Probes) was used at a concentration of 1 μM to stain extracellular DNA in biofilms [11]. TRITC (tetramethyl rhodamine isothiocyanate)-labeled wheat germ agglutinin (Molecular Probes, Eugene, OR) was used at a concentration of 0.1 mg/mL to stain the PIA in biofilms [17]. Hemoglobin was purchased from Sigma and used as indicated concentrations. The Ethics Committee of the Zhongshan Hospital of Fudan University and the East Hospital of Tongji University both exempted this study from review because the current study only focused on bacteria.

### Cultivation of bacterial biofilms

Biofilm cultivation in polystyrene microtitre plates was carried out as described previously [11]. Briefly, overnight cultures of *Se* strains grown in TSB (0.25% glucose) medium were diluted 1:200. The diluted cultures were transferred to wells of polystyrene microtitre plates (200 μL per well) and incubated at 37 °C for 24 h. After washing, the wells were stained with 2% crystal violet for 5 min. Then, the plate was rinsed, air-dried, redissolved in ethanol and the absorbance was determined at 590 nm. For cultivation of *Se* biofilms in the flow-chamber system, the flow-chamber system was first assembled and prepared as described previously [18]. Briefly, the flow chambers were inoculated by injecting 350 μL overnight culture diluted to OD600 = 0.001 into each flow channel with a small syringe. After inoculation, flow channels were left without flow for 1 h, after which medium flow (0.2 mm/s) was started using a Watson-Marlow 205 S peristaltic pump.

### Microscopy

All microscopic observations and image acquisition were performed using a Zeiss LSM 510 confocal laser scanning microscope (Carl Zeiss, Jena) equipped with detectors and filter sets for monitoring SYTO 9, PI, DDAO and TRITC fluorescence. Images were obtained using an x63/1.4i objective or an x40/1.3i objective. Simulated 3D images and sections were generated using the IMARIS software package (Bitplane).

### Bacterial attachment assays

Initial cell attachment was tested as described previously [11]. Briefly, cell suspensions from the mid-exponential phase of bacterial growth were diluted to OD600 = 0.1 in PBS, and then incubated in wells (1 mL per well) of cover-glass cell culture chambers (Nunc) for 30 min at 37°C, after which attached cells were calculated by microscopy.

### Quantification of extracellular DNA

Extracellular DNA was quantified as described previously [11]. Overnight cultures were diluted to OD600 = 0.001 in AB medium supplemented with 0.5% glucose, 0.05 mM PI and 10% TSB. The diluted cultures were transferred to wells of polystyrene microtitre plates (150 μL per well) and incubated for 24 h at 37°C, upon which PI absorbance was measured at 480 nm and cell density was measured by OD600 using a Wallac microtitre plate reader. Relative amounts of extracellular DNA per OD600 unit were calculated.

### qRT-PCR

After cultured for 1 d or 6 d, biofilm cells were resuspended in 500 mL buffer containing 25% sucrose, 10 mM Tris/HCl (pH 7.5) and 0.2 mg lysostaphin (Sigma-Aldrich). After incubation at 37°C for 10 min, total RNA was isolated using the RNeasy Mini kit according to the manufacturer’s instructions (QIAGEN). cDNA was synthesized from equivalent concentrations of total RNA using the SuperScript III First-Strand Synthesis SuperMix Kit (Invitrogen) according to the manufacturer’s instructions. Coding sequences for bacterial genes (and *gyrB* for internal controls) were amplified using iQ SYBR Green Supermix (Bio-rad). Custom primer sequences used for amplification experiments are included in Additional file 2: Table S1. Amplification was carried out using an iCycler IQ Real-Time PCR Detection System, and cycle threshold (Ct) values determined in duplicate for target gene transcripts and *gyrB* for each experiment. “No template” (water) and “no-RT” controls were used to ensure minimal background DNA contamination. Fold changes for experimental groups relative to assigned controls were calculated using automated iQ5 2.0 software (Bio-rad).

### PCR and sequencing

Genomic DNA was extracted by using Wizard Genomic DNA Purification Kit (Promega) according to the manufacturer’s instructions. The primers included in Additional file 2: Table S1 were designed from conserved sequences of *agr*, which are common to *agr* groups I, II and III, to amplify a 1022 bp fragment [19]. The PCR production was purified by using QIAquick PCR Purification Kit (Qiagen) then sequenced (Operon), and alignment analysis was performed by using Vector NTI Advance 9 software (Invitrogen).

### Cell autolysis assays

Autolysis assays for *Se* strains were performed as described previously [11]. Briefly, cell samples (50 mL) were collected from exponential-phase cultures growing in TSB medium (OD600 = 0.6 ~ 0.8) containing 1 M NaCl, and cells were pelleted by centrifugation. The cells were washed twice with 50 mL ice-cold water and resuspended in 50 mL 0.05 M Tris/HCl (pH 7.2) containing 0.05% (v/v) Triton X-100. The cells were then incubated at 30°C with shaking, and OD600 was measured at 30 min intervals. The lysis rate induced by Triton X-100 was calculated as: OD0-ODt/OD0.

## Results

### *Se* isolates associated with catheter infection exhibit more avid self-renewal in long-term cultured biofilm assays

**Figure 1:**
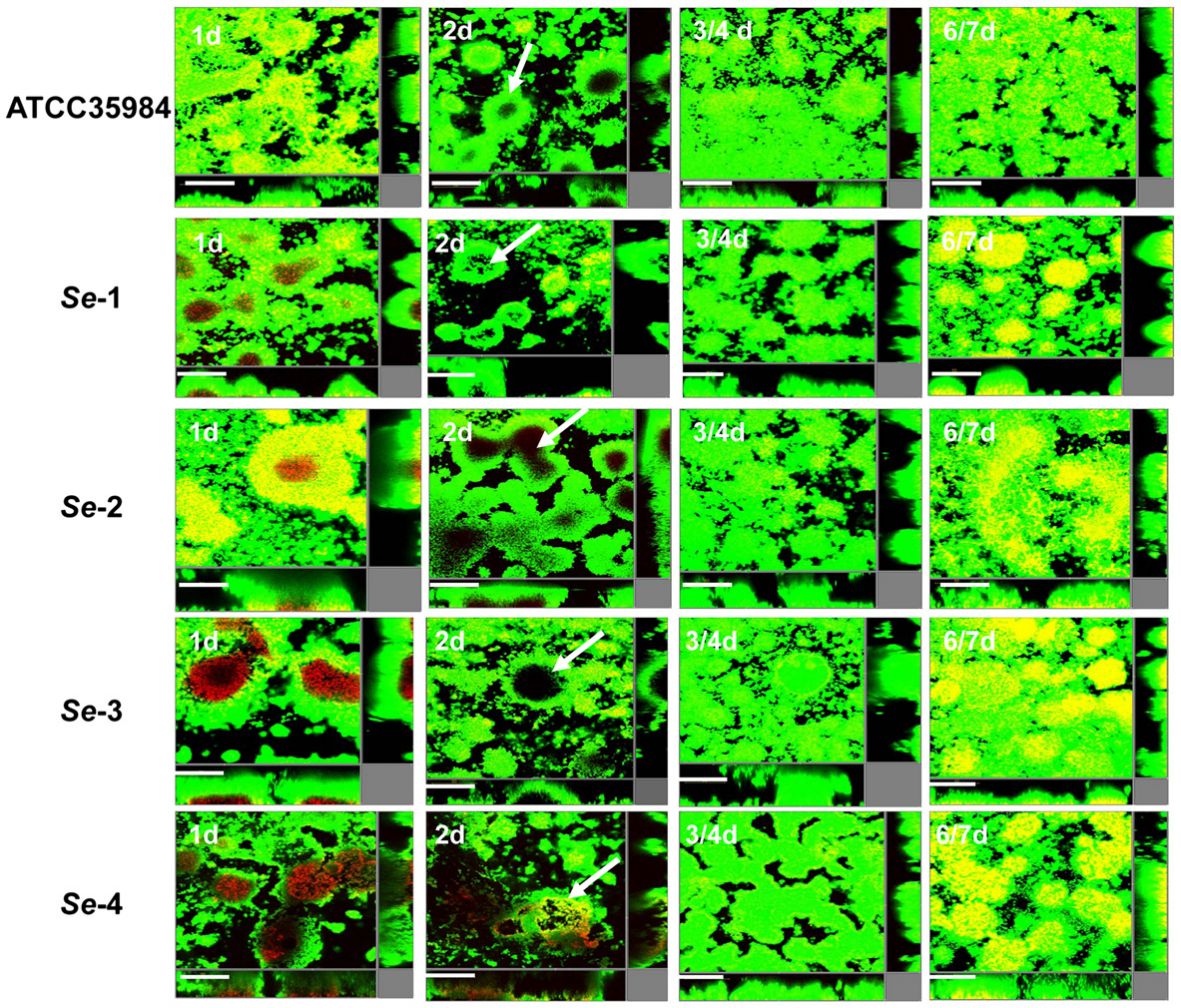
***S. epidermidis*isolates associated with catheter infection exhibit greater biofilm self-renewal.** Laboratory strain ATCC 35984 and clinical isolates *Se*-1, *Se*-2, *Se*-3 and *Se*-4 were grown for ~7 days in flow chambers irrigated with minimal medium, and stained with SYTO 9 and PI at indicated time points to identify live and dead cells, respectively. Microscopic investigation was performed using confocal laser scanning microscopy (CLSM). The central pictures show horizontal optical sections, and the flanking pictures show side views. Live cells appear green and dead cells appear yellow/red. Bars, 50 μm.

We first observed long-term (~7 days) cultured biofilm formation for *Se*-1-4 in the flow-chamber systems, together with one biofilm-positive *Se* reference strain (ATCC 35984). All strains displayed similar biofilm development during long-term cultivation, although they displayed heterogeneity for biofilm architecture (Figure 1). After one day in culture, the chamber surface was almost completely covered by bacterial biofilms, and many dead cells were present in the center of microcolonies. After 2 days, most of the dead cells were detached from the microcolonies, forming vacuoles. After 3-4 days, the residual cells in the old biofilms proliferated and occupied the vacuolated areas, forming a “renewed” biofilm in which only a few dead cells were observed. After 6-7 days, a large number of dead cells reappeared in the center of microcolonies. Notably, *Se*-1, *Se*-2, *Se*-3 and *Se*-4 displayed much bigger microcolonies, more dead cells, and more significant cell dispersal with much more vacuole formation relative to the reference strain ATCC 35984 (Figure 1).

### *Se* isolates associated with catheter infection exhibit greater extracellular DNA content and capacity for cell attachment

**Figure 2:**
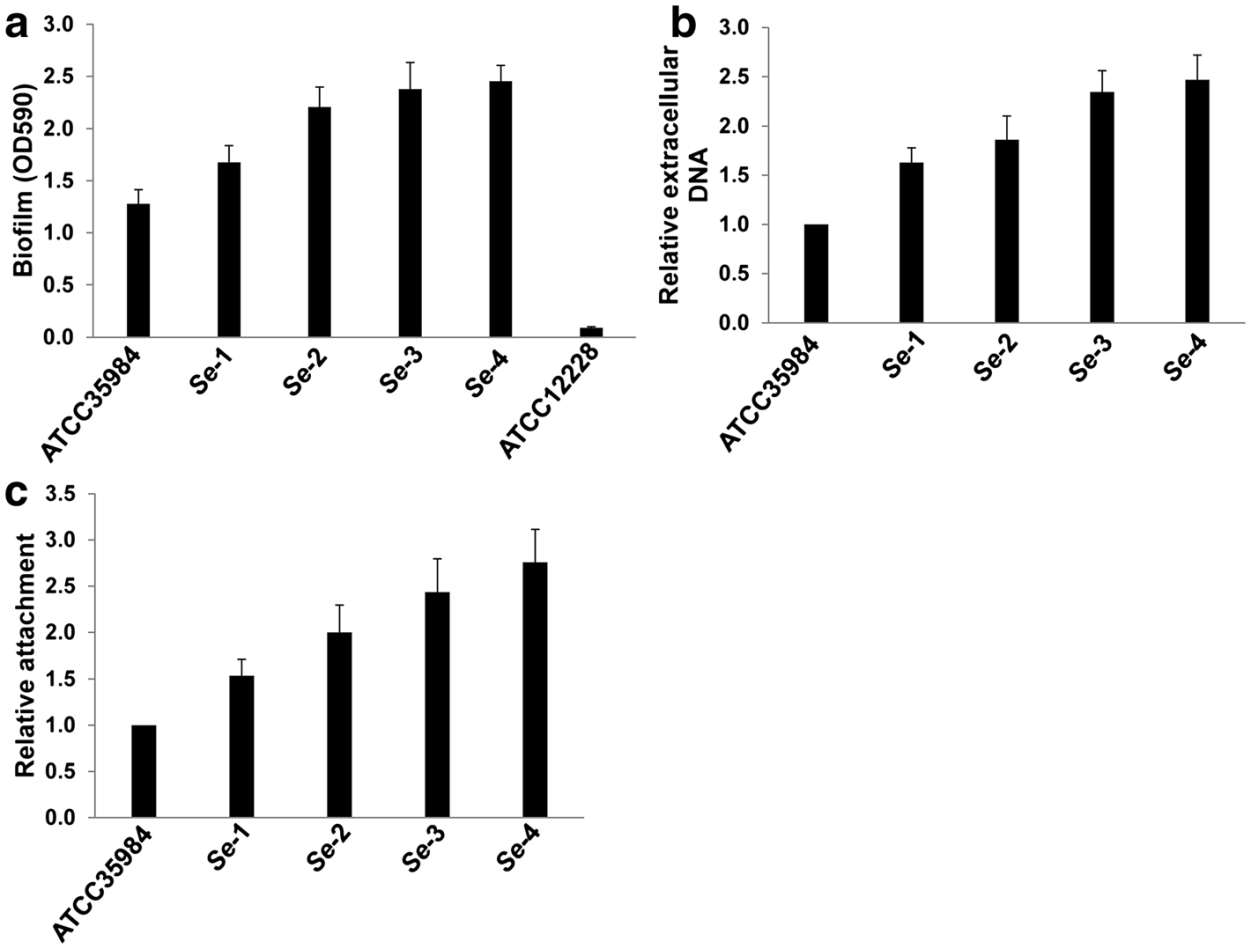
***S. epidermidis*isolates associated with catheter infection display more biofilm formation, extracellular DNA release and initial attachment than laboratory strain.(a)** Cultures were grown in microtitre plates for 24 h at 37°C, and biofilm biomass was quantified using a crystal violet assay. **(b)** Cultures were grown for 24 h in minimal medium supplemented with 0.05 mM PI, whereupon PI absorbance (OD480) and cell density (OD600) were measured and relative amounts of extracellular DNA per OD600 unit were calculated. **(c)** Initial attachment of *S. epidermidis* strains in static chambers was measured as described in Methods. Error bars represent the S.E.M. for three independent experiments.

We next compared biofilm formation capacity for these clinical isolates and the reference strain using the microtitre plates. These results first confirmed that all 4 *Se* clinical isolates displayed stronger biofilm biomass than ATCC 35984 by crystal violet staining (Figure 2A). Interestingly, we also found significantly more extracellular DNA release from these clinical isolates relative to the reference strain during biofilm formation (Figure 2B). Our previous study demonstrated that extracellular DNA is a major component required for initial bacterial attachment to surfaces, as well as subsequent early phases of biofilm development by *Se*[11]. In agreement with these results, we found that our clinical isolates exhibited a greater capacity for cell attachment relative to the reference strain (Figure 2C). PIA plays an important role in cell-cell adhesion during phase II of *Se* biofilm formation [10], and Jager et al. have previously reported detection of PIA synthesis in mature biofilms using TRITC-labeled wheat germ agglutinin staining [17]. However, we did not observe obvious differences in PIA synthesis between our *Se* clinical isolates and the reference strain (data not shown).

### The *agr* system regulates initial cell attachment and cell autolysis during biofilm formation for *se*

**Figure 3:**
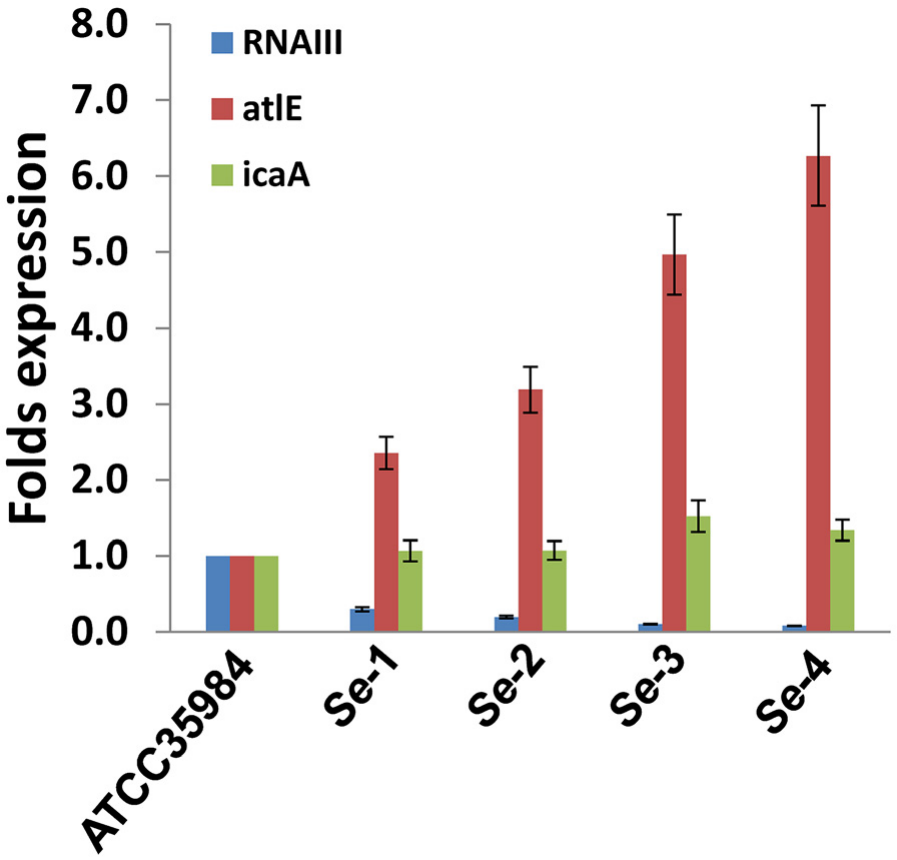
***S. epidermidis*isolates associated with catheter infection exhibit differential expression of genes associated with biofilm formation.** The expression profiles of *RNAIII*, *atlE* and *icaA* were compared for 24-h biofilm cells of laboratory strain and clinical isolates using qRT-PCR as described in Methods. Error bars represent the S.E.M. for three independent experiments.

**Figure 4:**
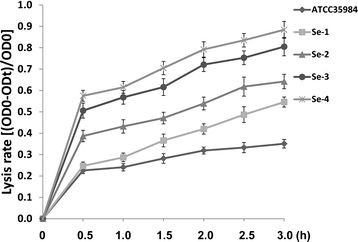
***S. epidermidis*isolates associated with catheter infection exhibit higher cell autolysis abilities.** Triton X-100 induced cell autolysis assays were performed as described in Methods, and error bars represent the S.E.M. for three independent experiments.

Next, we compared the biofilm-associated gene profiles for our *Se* clinical isolates and the reference strain by qRT-PCR. Our results indicated that expression of *atlE*, the major autolysin gene of *Se* required for initial cell attachment, extracellular DNA release and Triton X-100 induced autolysis [7,11,13], was significantly increased in all the 4 clinical isolates (~2-7 fold) relative to the reference strain for 1 d- or 6 d-biofilm cells (Figure 3, Additional file 3: Figure S2). In contrast, there were no appreciable differences for expression of *icaA*, the gene encoding N-acetylglucosaminyltransferase and required for PIA synthesis and cell-cell aggregation among them. Notably, expression of *RNAIII*, a gene encoding an effector molecule of the *agr* quorum sensing system, was significantly reduced for all the *Se* clinical isolates relative to the reference strain (Figures 3, Additional file 3: Figure S2). Further experiments revealed that all the 4 clinical isolates displayed stronger cell autolysis abilities than ATCC35984 induced by Triton X-100 (Figure 4).

### *Agr* mutant increases initial cell attachment and cell death during biofilm formation through upregulation of *atlE*

**Figure 5:**
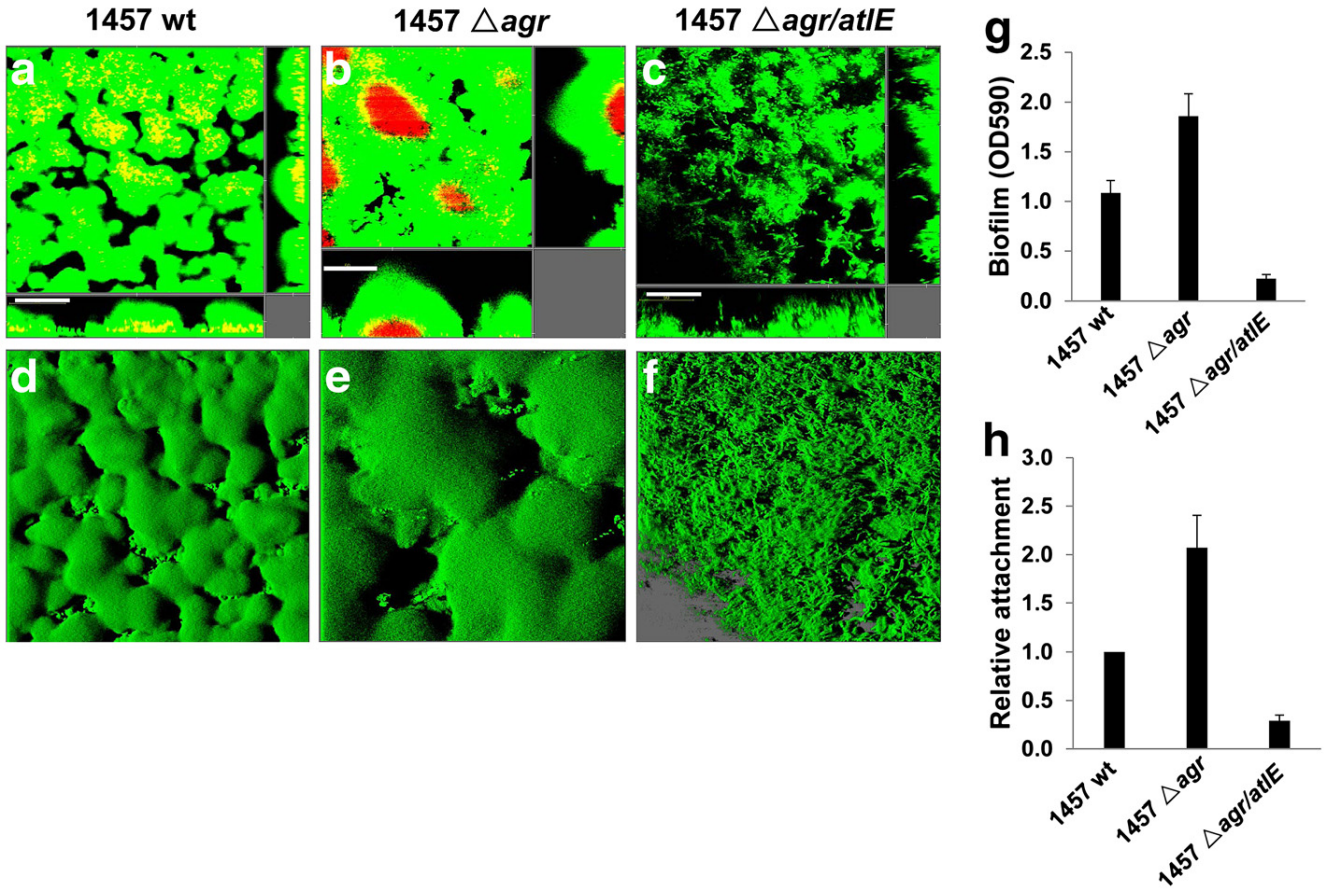
***S. epidermidis agr*system regulates biofilm formation and initial cell attachment through*atlE*.** ( **a-d**) *S. epidermidis* 1457 wild type (wt, **a** and **d**), *agr* mutant (△ *agr*, **b** and **e**) and *agr/atlE* double mutant (△ *agr/atlE*, **c** and **f**) were grown for 24 h in flow chambers irrigated with minimal medium, and were then stained with SYTO 9 and PI, upon which microscopic investigation was performed by CLSM. The 3-D images **(d-f)** were generated using the IMARIS, bars, 50 μm. **(g)** Biofilm biomass in microtitre plates was quantified using a crystal violet assay. **(h)** Initial attachment of *S. epidermidis* strains in static chambers was quantified as described in Methods. Error bars represent the S.E.M. for three independent experiments.

To further clarify the roles of *agr* in cell attachment, cell death and biofilm formation, we assessed these endpoints for *Se* 1457 wild type (wt), *agr* mutant *(△agr*) and *agr*/ *atlE* double mutant *(△agr/atlE*) strains using our flow-chamber systems. We found more dead cells in the center of microcolony structures for 1457 *△agr* mature biofilms than 1457 wt (Figure 5A, B), while only few dead cells were seen in 1457 *△agr/atlE* (Figure 5C). Also, 1457 *△agr* displayed thicker microcolony structure during biofilm formation than 1457 wt (Figure 5D, E), in contrast, the biofilm formation ability of 1457 *△agr/atlE* was seriously impaired because it only formed very thin and loose biofilm structure (Figure 5F). Of note, cell dispersal, vacuole formation, and self-renewal biofilms were also observed after long-term culture in flow-chamber systems (data not shown). Crystal violet staining further confirmed that 1457 *△agr* formed stronger biomass than 1457 wt in the microtitre plate assays, while 1457 *△agr/atlE* only formed poor biomass (Figure 5G). Finally, 1457 *△agr* exhibited significantly increased initial cell attachment relative to 1457 wt, whereas which was impaired in 1457 *△agr/atlE* (Figure 5H).

### *Agr* regulates *se* release of extracellular DNA and autolysis through suppression of *atlE*

**Figure 6:**
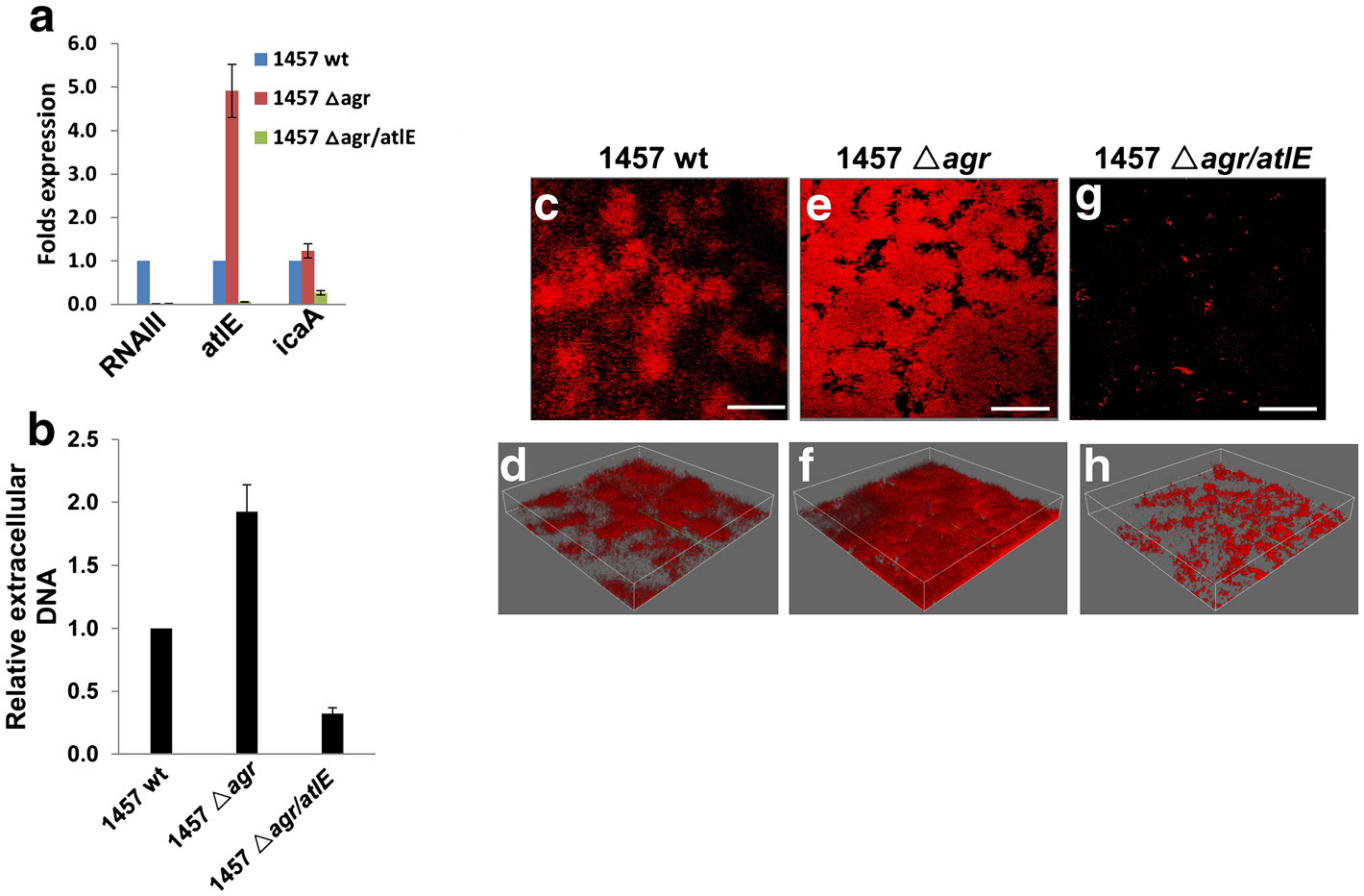
***S. epidermidis agr*system controls extracellular DNA release through*atlE*.(a)** Biofilm-associated gene transcripts were compared between 1457 wt, △ *agr* and △ *agr/atlE* by using qRT-PCR. **(b)** Extracellular DNA release from cultures in microtitre plates was quantified as described above. Error bars represent the S.E.M. for three independent experiments. **(c-h)***S. epidermidis* 1457 wild type (wt, **c-d**) *agr* mutant (△ *agr*, **e** and **f**) and *agr/atlE* double mutant (△ *agr/atlE*, **g** and **h**) were grown for 24 h in flow chambers irrigated with minimal medium, and were then stained with DDAO for extracellular DNA in biofilms, upon which microscopic investigation was performed by CLSM. The 3-D images ( **d**/ **f**/ **h**) were generated using the IMARIS, bars, 50 μm.

Our previous study revealed that mutation of *atlE* in *Se* 1457 significantly reduced extracellular DNA release and impairs biofilm formation [11]. Consistent with those results, qRT-PCR revealed that expression of *atlE* was significantly increased for 1457 *△agr*, but almost no *atlE* transcripts were detected in 1457 *△agr/atlE* (Figure 6A). Our qRT-PCR also confirmed that no *RNAIII* transcripts were detected in *Se* 1457 *△agr*, when compared with its wt strain (Figure 6A). Furthermore, 1457 *△agr* exhibited increased extracellular DNA relative to 1457 wt using both microtitre plate assays and DDAO staining in the flow-chamber systems (Figure 6C-F), while 1457 *△agr/atlE* abolished most extracellular DNA (Figure 6B6G-H). In addition, 1457 *△agr* displayed higher cell autolysis abilities than its wt strain, when induced by Triton X-100, whereas poor cell autolysis was seen in 1457 *△agr/atlE* (Additional file 4: Figure S3). Notably, expression of *icaA* transcripts was almost unchanged for 1457 *△agr* relative to its wt strain, however, *icaA* transcripts were partially reduced in 1457 *△agr/atlE* (Figure 6A).

### Chemical inhibition of *agr* increases biofilm formation, initial attachment and cell autolysis through upregulation of *atlE*

**Figure 7:**
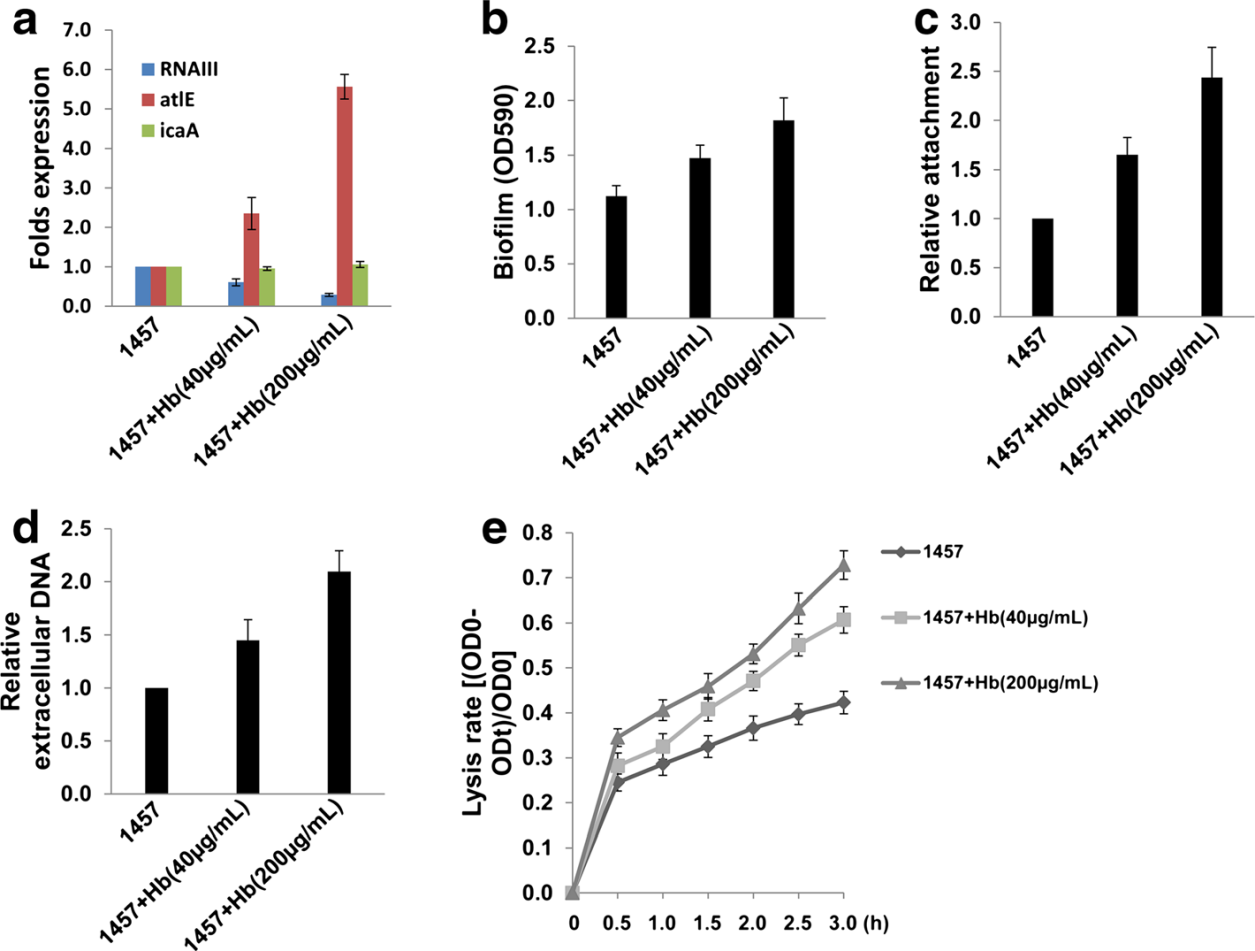
**Chemical inhibition of*agr*exhibit increased biofilm formation, extracellular DNA release and cell autolysis through upregulation of*atlE*.***S. epidermidis* 1457 was treated with or without hemoglobin (40 or 200 μg/mL), then **(a)** Biofilm-associated gene transcripts were measured by using qRT-PCR; **(b)** Biofilm biomass was quantified using a crystal violet assay; **(c-e)** Initial attachment, extracellular DNA release and cell autolysis were determined as described above, respectively. Error bars represent the S.E.M. for three independent experiments.

A recent study has revealed that inhibition of *S. aureus agr* system by human hemoglobin promotes surface colonization [20]. To confirm the roles of *agr* in biofilm-associated events we found in *Se* 1457 genetic mutants above, here we treated *Se* 1457 wt strain with or without human hemoglobin (40 or 200 μg/mL). The results indicated that hemoglobin significantly reduced *RNAIII* transcripts (~40%-70% of inhibition) while increased *atlE* (~2.5-5.5 folds) but almost not affecting *icaA* (Figure 7). Functional assays further confirmed that hemoglobin increased biofilm formation, initial attachment, extracellular DNA release and cell autolysis in a dose-dependent manner (Figure 7), while which does not affect bacterial growth (data not shown).

## Discussion

*Se* biofilm formation on implanted medical devices may result in recurrent or refractory infection unless the devices are removed, and removal and replacement of these devices incurs significant cost and risk for the patient. Flow-chamber systems simulate blood or other body-fluid flow in the vasculature of patients [18]. Using this and other complimentary approaches, we found that clinical *Se* isolates from patients with implanted catheter infections display greater microcolony densities, spontaneous cell death, and self-renewal capacity during biofilm development relative to reference strains. Bacteria in biofilms are 100 ~ 1000 times more resistant to antibiotics than planktonic cells [21-23], although our study does not directly address antibiotic sensitivity for our clinical isolates. Staphylococcal biofilm dispersal is associated with severe infection, including endocarditis, pneumonia and sepsis [24-26]. In addition, dispersal cells help bacteria establish new biofilms in more suitable niches, resulting in infection within multiple tissues [27]. Of interest, we collected the detached and “flow-out” cells in the flow-chamber systems for our clinical isolates and found living cells capable of forming new biofilms as quickly as their parent cells (Qin et al., unpublished data).

Interestingly, expression of *RNAIII*, a gene for the effector molecule of the *agr* system, was significantly reduced in all 4 *Se* clinical isolates, suggesting that the functions of *agr* quorum-sensing system were impaired in these isolates. Besides its regulatory function, *RNAIII* also encodes a δ-toxin, which effectively reduces cell attachment and subsequent biofilm formation of a *Se agr* mutant [13]. Our work does not address how *RNAIII* transcripts might be downregulated in our clinical isolates. Ongoing studies have found some potential spontaneous mutations present in the *agr* conserved region from *Se*-1, *Se*-2, *Se*-3 when compared with ATCC35984 strain (see Additional file 5: Figure S4), while *Se*-4 displayed some larger fragment variations for unknown reasons (Qin et al., unpublished data). However, it still requires further investigations to identify these potential spontaneous mutations responsible for *RNAIII* transcripts downregulation in these clinical isolates. Interestingly, about ~25% of *S. aureus* and ~17% of *Se* clinical isolates are naturally occurring *agr* mutants [19,28]. One recent study indicated that *Se agr* mutant showed increased biofilm development and colonization in a rabbit model [29]. In addition, nonfunctional *agr* occurred more frequently among strains isolated from infections of joint prostheses, which includes some mutations caused by insertion of an IS256 element [29]. Moreover, polymorphisms within the *agr* locus for staphylococci are associated with its pathogenicity [19,29,30]. We have also observed that *agr*-positive (with normal *RNAIII* transcription) *Se* clinical isolates retain capacity for self-renewal in long-term culture (Qin et al., unpublished data), suggesting that other mechanisms are responsible for self-renewal for these isolates. Another recent study reported that addition of a cyclic autoinducing peptide (AIP) to activate *agr* in *S. aureus agr*–positive strains mediated dramatic detachment of *S. aureus* biofilms through an increase in expression of Aur metalloprotease and the SplABCDEF serine proteases [31]. However, it is unclear whether these proteases may have similar functions in biofilms formed by *agr*–positive *Se* strains.

Expression of the gene encoding autolysin, *atlE*, was significantly increased in all 4 our clinical isolates. Previous data indicate that *atlE* expression is essential for initial cell attachment and biofilm formation by *Se*[7,11,13]. We previously reported that isogenic deletion of *atlE* in *Se* 1457 significantly reduced cell attachment, extracellular DNA release, cell autolysis and final biofilm formation [11]. We and others found that *atlE* transcripts were significantly increased in *Se* 1457 *agr* mutants, which exhibited enhanced cell attachment, extracellular DNA release, cell death ( *atlE*-mediated autolysis) and subsequent biofilm formation [13]. In contrast, we found that *Se* 1457 *agr/atlE* double mutant seriously impaired these features mentioned above in the current study. In fact, we think that increased densities of microcolonies in *Se* mutant mature biofilms will cause more cell death and detachment due to nutrition deficiency, oxygen stress or other reasons required further investigation. In addition, other mechanisms have also been recently reported to be related with staphylococcal extracellular DNA release and biofilm dissemination, including the *cidA* murein hydrolase regulator [32] and the β subclass of phenol-soluble modulins (PSMs) [26].

## Conclusion

Taken together, our studies illuminate a novel mechanism for enhancing *Se* biofilm self-renewal: repression of *agr* and induction of *atlE* expression, which results in increased cell autolysis/death, biofilm dispersal and final promoting new biofilm formation. Better understanding the process and mechanisms of *Se* biofilm self-renewal in patients will help us develop more effective strategies against *Se* biofilm-related infection.

## Competing interests

All authors declare that they have no competing interests.

## Authors’ contributions

Conceived and designed the experiments: LY, ZQ and SM. Performed the experiments: LD, LY, VJF and ZQ. Analyzed the data: LD and ZQ. Contributed reagents/materials/analysis tools: VJF, CP and SM. Wrote the manuscript: CP, SM and ZQ. All authors read and approved the final manuscript.

## Supplementary Material

Additional file 1 **Figure S1.***S. epidermidis* 1457 *agr* mutation does not affect bacterial growth. Growth curves for *S. epidermidis* 1457 wild type and *agr* mutant and *agr/atlE* double mutant cultivated in TSB batch cultures are shown. Data shown represent one of 3 independent experiments. Click here for file

Additional file 2 **Figure S2.***S. epidermidis* isolates associated with catheter infection exhibit differential expression of genes associated with biofilm formation. The expression profiles of *RNAIII*, *atlE* and *icaA* were compared for 6-d biofilm cells of laboratory strain and clinical isolates using qRT-PCR as described in Methods. Error bars represent the S.E.M. for three independent experiments. Click here for file

Additional file 3 **Figure S3.***S. epidermidis agr* system regulates cell autolysis through *atlE*. Triton X-100 induced cell autolysis assays were performed as described in Methods, and error bars represent the S.E.M. for three independent experiments. Click here for file

Additional file 4 **Figure S4.** Sequence alignment analysis of *agr* conserved regions from ATCC 35984, *Se*-1, *Se*-2 and *Se*-3. The *agr* conserved regions were amplified and sequenced as described in Methods, then alignment analysis was performed by using Vector NTI Advance 9 software (Invitrogen). Click here for file

Additional file 5 **Table S1.** Primer sequences for qRT-PCR in this study. Click here for file
