# Supplementary figures and images for: Staphylococcus epidermidis recovered from indwelling catheters exhibit enhanced biofilm dispersal and “self-renewal” through downregulation of agr
Source: BMC Microbiol. 2012 Jun 8;12:102. doi: 10.1186/1471-2180-12-102 (PMC3458918; doi:10.1186/1471-2180-12-102)

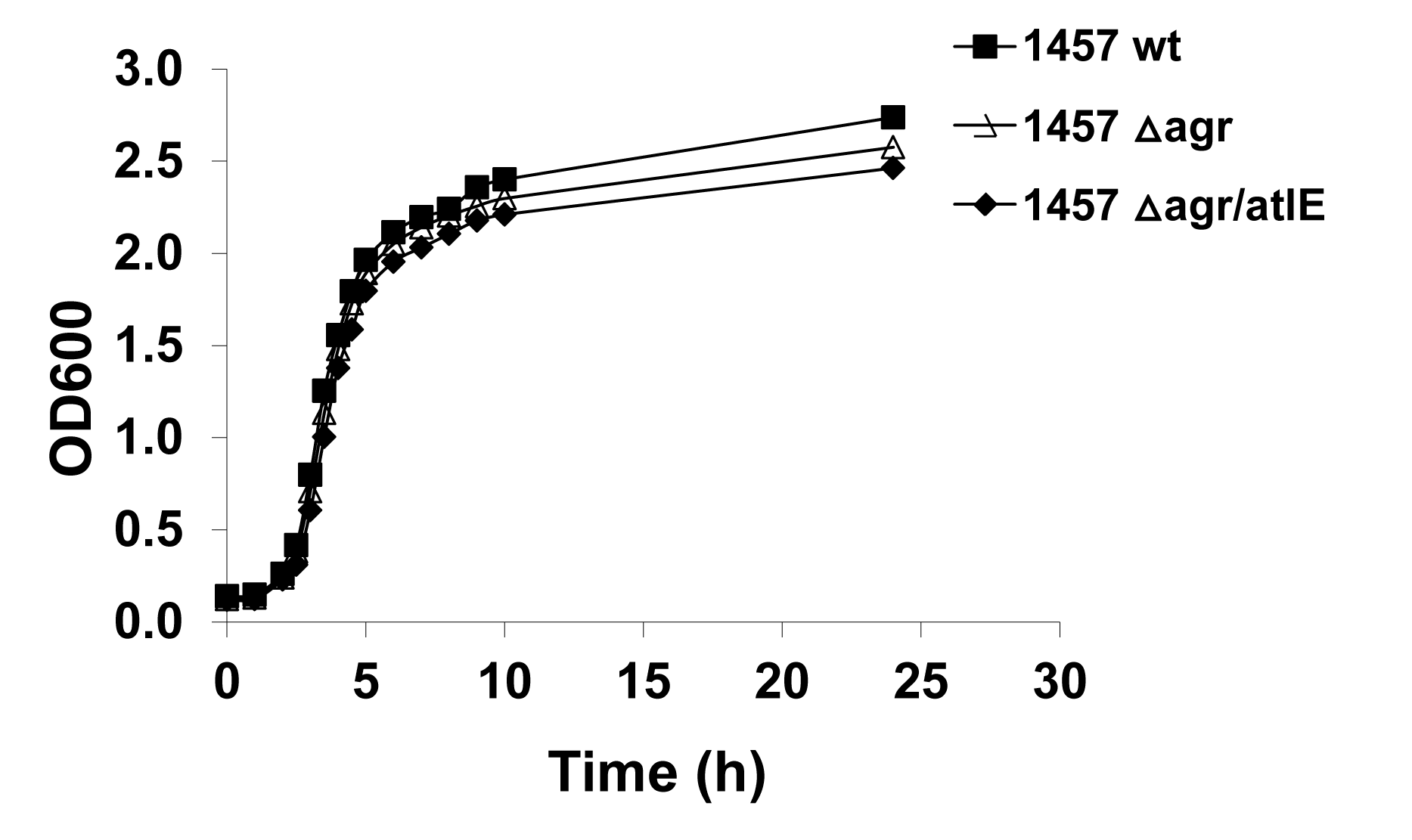

Supplement: Additional file 1 — Figure S1.S. epidermidis 1457 agr mutation does not affect bacterial growth. Growth curves for S. epidermidis 1457 wild type and agr mutant and agr/atlE double mutant cultivated in TSB batch cultures are shown. Data shown represent one of 3 independent experiments. [file 1471-2180-12-102-S1.tiff]

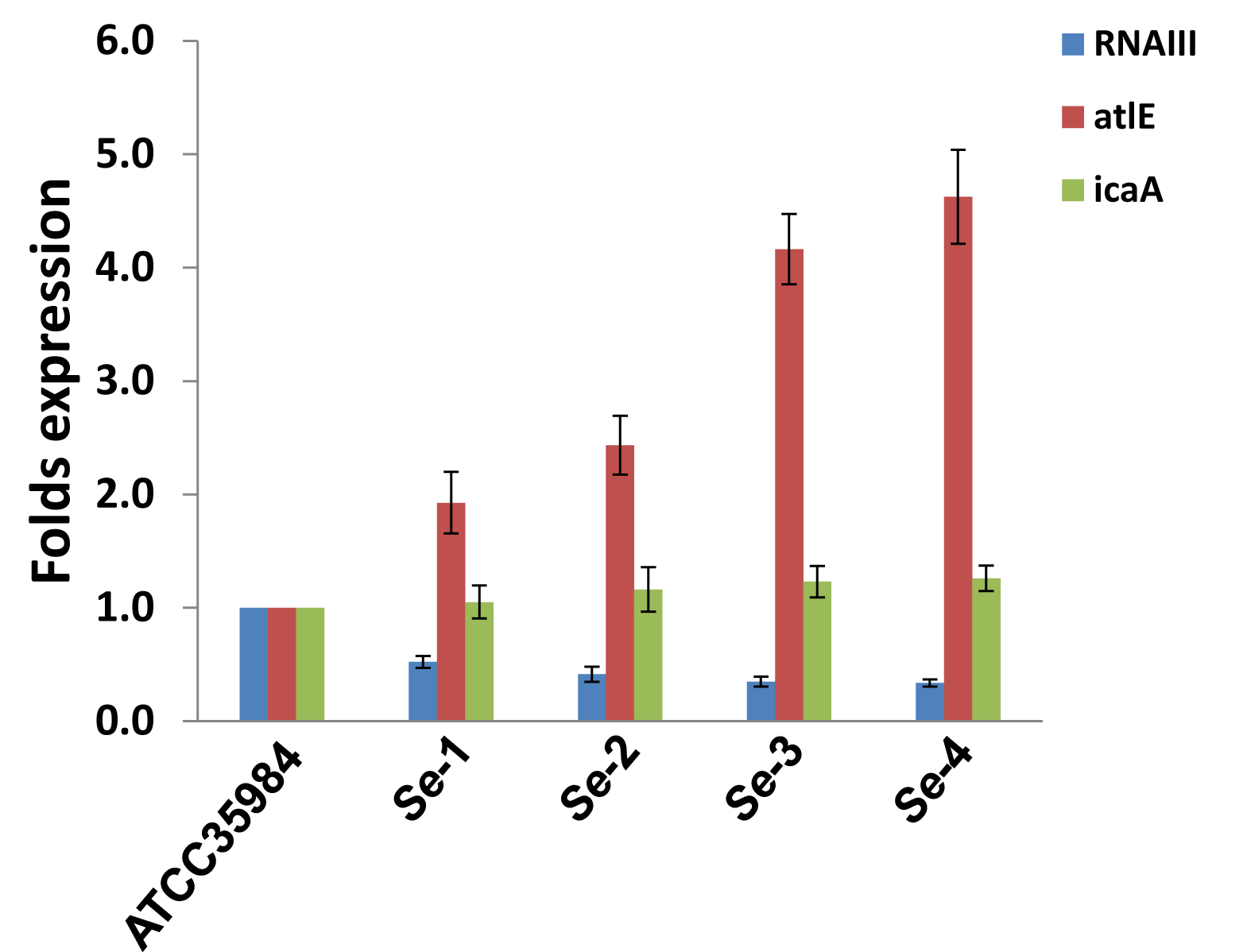

Supplement: Additional file 2 — Figure S2.S. epidermidis isolates associated with catheter infection exhibit differential expression of genes associated with biofilm formation. The expression profiles of RNAIII, atlE and icaA were compared for 6-d biofilm cells of laboratory strain and clinical isolates using qRT-PCR as described in Methods. Error bars represent the S.E.M. for three independent experiments. [file 1471-2180-12-102-S2.tiff]

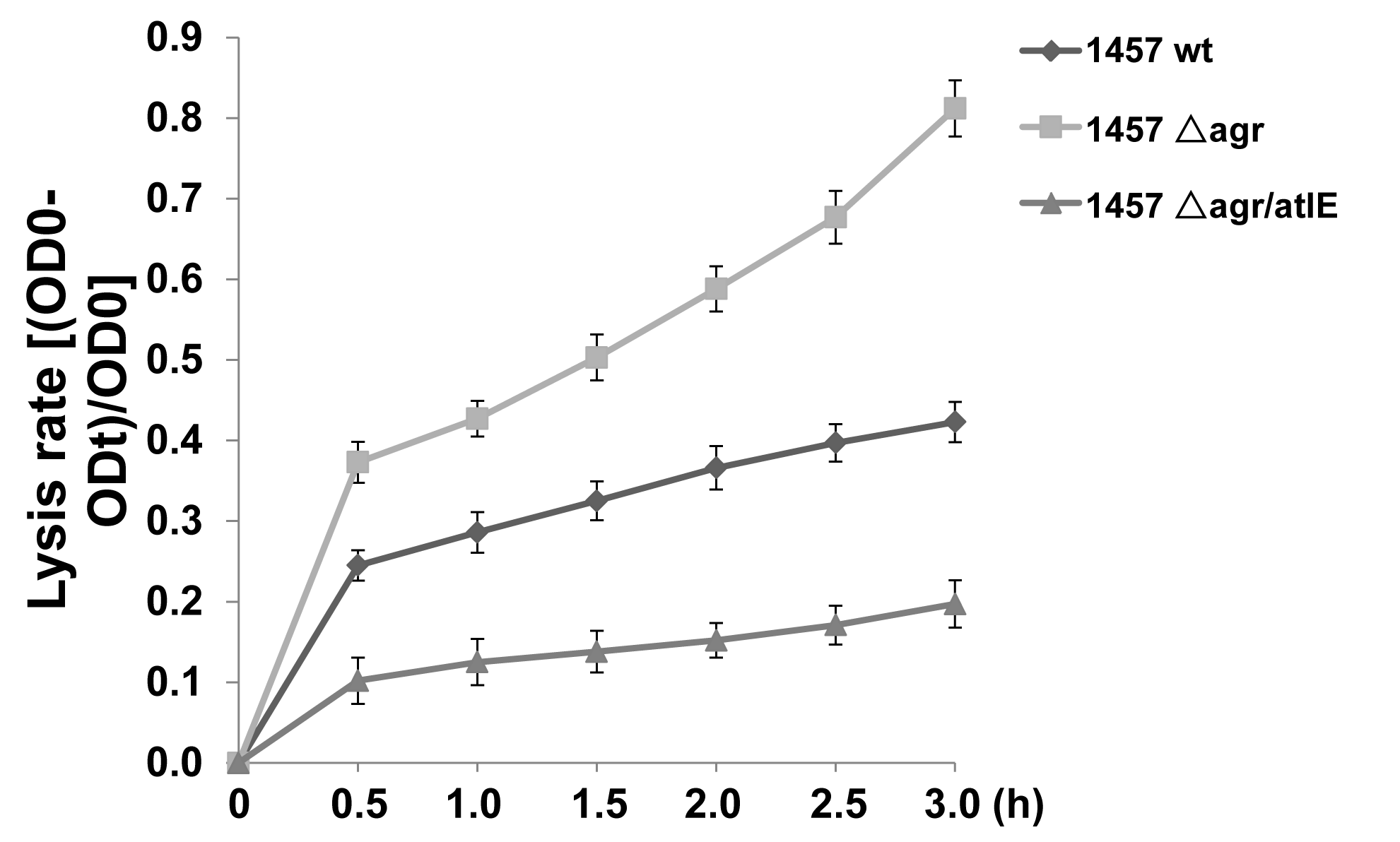

Supplement: Additional file 3 — Figure S3.S. epidermidis agr system regulates cell autolysis through atlE. Triton X-100 induced cell autolysis assays were performed as described in Methods, and error bars represent the S.E.M. for three independent experiments. [file 1471-2180-12-102-S3.tiff]
