# Supplementary material for: Staphylococcus epidermidis recovered from indwelling catheters exhibit enhanced biofilm dispersal and “self-renewal” through downregulation of agr
Source: BMC Microbiol. 2012 Jun 8;12:102. doi: 10.1186/1471-2180-12-102 (PMC3458918; doi:10.1186/1471-2180-12-102)
Supplement: Additional file 4 — Figure S4. Sequence alignment analysis of agr conserved regions from ATCC 35984, Se-1, Se-2 and Se-3. The agr conserved regions were amplified and sequenced as described in Methods, then alignment analysis was performed by using Vector NTI Advance 9 software (Invitrogen). [file 1471-2180-12-102-S4.pdf]

Untitled

```
                                                                  Section 1
               (1)  1                 10                  20                  30                  40                51
ATCC35984_Agr  (1)  ---NNNNNNNNNNNNAGCGAAANTACTCTCCATATTTTTA-TATTGTACTA
     Se-2_Agr  (1)  --NNNNNNNNNNNNAAGCGANNNN-CTCTCCATATTTTTA-TATTGTACTA
     Se-1_Agr  (1)  NNNNNNNNNNNNNNAAGCGAAAATACTCTCCATATTTTTA-TATTGTACTA
     Se-3_Agr  (1)  -NNNNNNNNNNNNNAAGCGNNNNNACNCNNCNTATTTTNNANATTGTACTN
    Consensus  (1)   NNNNNNNNNNNNNAAGCGANNNTACTCTCCATATTTTTA TATTGTACTA
                                                                  Section 2
              (52)  52              60                  70                  80                  90                102
ATCC35984_Agr (48)  -TCGTAGTTATTTCATTAGTAACTAAAGAACCGGTAAATAAACTTATTTTA
     Se-2_Agr (48)  -TCGTAGTTATTTCATTAGTAACTAAAGAACCGGTAAATAAACTTATTTTA
     Se-1_Agr (51)  -TCGTAGTTATTTCATTAGTAACTAAAGAACCGGTAAATAAACTTATTTTA
     Se-3_Agr (51)  ATCGTAGTTATTTCATTAGTAACTAAAGAACCGGTAAATAAACTTATTTTA
    Consensus (52)   TCGTAGTTATTTCATTAGTAACTAAAGAACCGGTAAATAAACTTATTTTA
                                                                  Section 3
             (103)  103           110                 120                 130                 140               153
ATCC35984_Agr (98)  TTCGGTGTAATTTTAGAATCTTTAACATTACTACCCATCTTTTTCCCTAAG
     Se-2_Agr (98)  TTCGGTGTAATTTTAGAATCTTTAACATTACTACCCATCTTTTTCCCTAAG
     Se-1_Agr (101) TTCGGTGTAATTTTAGAATCTTTAACATTACTACCCATCTTTTTCCCTAAG
     Se-3_Agr (102) TTCGGTGTAATTTTAGAATCTTTAACATTACTACCCATCTTTTTCCCTAAG
    Consensus (103) TTCGGTGTAATTTTAGAATCTTTAACATTACTACCCATCTTTTTCCCTAAG
                                                                  Section 4
             (154)  154         160                 170                 180                 190               204
ATCC35984_Agr (149) GAGGATATTAATCATGGAAAACATTTTTAATTTATTTATAAAATTTTTCAC
     Se-2_Agr (149) GAGGATATTAATCATGGAAAACATTTTTAATTTATTTATAAAATTTTTCAC
     Se-1_Agr (152) GAGGATATTAATCATGGAAAACATTTTTAATTTATTTATAAAATTTTTCAC
     Se-3_Agr (153) GAGGATATTAATCATGGAAAACATTTTTAATTTATTTATAAAATTTTTCAC
    Consensus (154) GAGGATATTAATCATGGAAAACATTTTTAATTTATTTATAAAATTTTTCAC
                                                                  Section 5
             (205)  205       210                 220                 230                 240                 255
ATCC35984_Agr (200) TACAATCTTGGAATTTATTGGTACTGTAGCAGGAGATAGTGTATGTGCCTC
     Se-2_Agr (200) TACAATCTTGGAATTTATTGGTACTGTAGCAGGAGATAGTGTATGTGCTTC
     Se-1_Agr (203) TACAATCTTGGAATTTATTGGTACTGTAGCAGGAGATAGTGTATGTGCTTC
     Se-3_Agr (204) TACAATCTTGGAATTTATTGGTACTGTAGCAGGAGATAGTGTATGTGCTTC
    Consensus (205) TACAATCTTGGAATTTATTGGTACTGTAGCAGGAGATAGTGTATGTGCTTC
                                                                  Section 6
             (256)  256                           270                 280                 290               306
ATCC35984_Agr (251) TTACTTTGACGAACCAGAAGTACCAGAAGAACTGACTAAACTATACGAGTA
     Se-2_Agr (251) TTACTTTGACGAACCAGAAGTGCCAGAAGAACTGACTAAACTATACGAGTA
     Se-1_Agr (254) TTACTTTGACGAACCAGAAGTGCCAGAAGAACTGACTAAACTATACGAGTA
     Se-3_Agr (255) TTACTTTGACGAACCAGAAGTGCCAGAAGAACTGACTAAACTATACGAGTA
    Consensus (256) TTACTTTGACGAACCAGAAGTGCCAGAAGAACTGACTAAACTATACGAGTA
                                                                  Section 7
             (307)  307                       320                 330                 340                   357
ATCC35984_Agr (302) AATATAACCCTAGAAAGTGTGTAAGATATGGATGATATTAATCTATTTCCG
     Se-2_Agr (302) AATATAACCCTAGAAAGTGTGTAAGATATGGATGATATTAATTTATTTCCG
     Se-1_Agr (305) AATATAACCCTAGAAAGTGTGTAAGATATGGATGATATTAATTTATTTCCG
     Se-3_Agr (306) AATATAACCCTAGAAAGTGTGTAAGATATGGATGATATTAATTTATTTCCG
    Consensus (307) AATATAACCCTAGAAAGTGTGTAAGATATGGATGATATTAATTTATTTCCG
```

Section 8

```
               (358) 358        370       380       390                 408
ATCC35984_Agr  (353) TTTGCAGGCCTACAAATCTTTTTAATGATTTGGGTTACTAAAGTTATCATT
     Se-2_Agr  (353) TTTGCAGGCCTACAAATCTTTTTAATGATTTGGGTTACTAAAGTTATCATT
     Se-1_Agr  (356) TTTGCAGGCCTACAAATCTTTTTAATGATTTGGGTTACTAAAGTTATCATT
     Se-3_Agr  (357) TTTGCAGGCCTACAAATCTTTTTAATGATTTGGGTTACTAAAGTTATCATT
    Consensus  (358) TTTGCAGGCCTACAAATCTTTTTAATGATTTGGGTTACTAAAGTTATCATT
```

Section 9

```
               (409) 409       420       430       440                 459
ATCC35984_Agr  (404) AATATGAAATTTAATTTTAGGGATTACATAATCGTTTTTACGATTGTAATC
     Se-2_Agr  (404) AATATGAAATTTAATTTTAGGGATTACATAATCGTTTTTACGATTGTAATC
     Se-1_Agr  (407) AATATGAAATTTAATTTTAGGGATTACATAATCGTTTTTACGATTGTAATC
     Se-3_Agr  (408) AATATGAAATTTAATTTTAGGGATTACATAATCGTTTTTACGATTGTAATC
    Consensus  (409) AATATGAAATTTAATTTTAGGGATTACATAATCGTTTTTACGATTGTAATC
```

Section 10

```
               (460) 460       470       480       490       500       510
ATCC35984_Agr  (455) CCTTCTGCAATAATGTATTACTTTTGGCAAAGTAAAGCATTAATAGTTTTG
     Se-2_Agr  (455) CCTTCTGCTATAATGTATTACTTTTGGCAAAGTAAAGCATTAATAGTTTTG
     Se-1_Agr  (458) CCTTCTGCTATAATGTATTACTTTTGGCAAAGTAAAGCATTAATAGTTTTG
     Se-3_Agr  (459) CCTTCTGCTATAATGTATTACTTTTGGCAAAGTAAAGCATTAATAGTTTTG
    Consensus  (460) CCTTCTGCTATAATGTATTACTTTTGGCAAAGTAAAGCATTAATAGTTTTG
```

Section 11

```
               (511) 511      520       530       540       550         561
ATCC35984_Agr  (506) GTTATAATAATCACCATTTTCTTTTATACAAAAATAAAGCTTTATTCAATA
     Se-2_Agr  (506) GTTATAATAATCATAATTTTCTTTTATACAAAAATAAAACTTTATTCAATA
     Se-1_Agr  (509) GTTATAATAATCATAATTTTCTTTTATACAAAAATAAAACTTTATTCAATA
     Se-3_Agr  (510) GTTATAATAATCATAATTTTCTTTTATACAAAAATAAAACTTTATTCAATA
    Consensus  (511) GTTATAATAATCATAATTTTCTTTTATACAAAAATAAAACTTTATTCAATA
```

Section 12

```
               (562) 562     570       580       590       600         612
ATCC35984_Agr  (557) TTAGTTGTATTATTCACCACTATGATCTTATATATAACTAATTTCATAACT
     Se-2_Agr  (557) TTAGTTGTATTATTCACGACTATGATCTTATATATAACTAATTTCATAACT
     Se-1_Agr  (560) TTAGTTGTATTATTCACGACTATGATCTTATATATAACTAATTTCATAACT
     Se-3_Agr  (561) TTAGTTGTATTATTCACGACTATGATCTTATATATAACTAATTTCATAACT
    Consensus  (562) TTAGTTGTATTATTCACGACTATGATCTTATATATAACTAATTTCATAACT
```

Section 13

```
               (613) 613    620       630       640       650          663
ATCC35984_Agr  (608) GTATACATACATTTGACTATAAAAGATTATATTCCGTTTAAATTTGCTTTA
     Se-2_Agr  (608) GTATACATACATTTAACTATAAAAGATTATATTCCGTTTAAATTTGTTTTA
     Se-1_Agr  (611) GTATACATACATTTAACTATAAAAGATTATATTCCGTTTAAATTTGTTTTA
     Se-3_Agr  (612) GTATACATACATTTAACTATAAAAGATTATATTCCGTTTAAATTTGTTTTA
    Consensus  (613) GTATACATACATTTAACTATAAAAGATTATATTCCGTTTAAATTTGTTTTA
```

Section 14

```
               (664) 664    670       680       690       700          714
ATCC35984_Agr  (659) CAGTTAATACATTTTACCTCTTTTGTAATCATAACTCNN-TTATTGCTTAT
     Se-2_Agr  (659) CAGTTAATACATTTTACCTTTTTTGTAATCATAACTCNN-TCATTGCTTAT
     Se-1_Agr  (662) CAGTTAATACATTTTACCTTTTTTGTAATCATAACTCTNATCATTGCTTAT
     Se-3_Agr  (663) CAGTTAATACATTTTACCTTTTTTGTAATCATAACTCTAATCATTGCTTAT
    Consensus  (664) CAGTTAATACATTTTACCTTTTTTGTAATCATAACTCTNATCATTGCTTAT
```

```
                                                                  Section 15
                   (715) 715  720       730       740       750            765
ATCC35984_Agr      (709) TTAACTCAACTATNGTTCAATAAATTAAAAGTATCCTACTTGTCACTCAAT
Se-2_Agr           (709) TTAACTCAACTATNGTTCAATAAATTAAAAGTATCCTATCTGTCACTCAAT
Se-1_Agr           (713) TTAACTCAACTATTGTTCAATAAATTAAAAGTATCCTATCTGTCACTCAAT
Se-3_Agr           (714) TTAACTCAACTATTGTTCAATAAATTAAAAGTATCCTATCTGTCACTCAAT
Consensus          (715) TTAACTCAACTATTGTTCAATAAATTAAAAGTATCCTATCTGTCACTCAAT
                                                                  Section 16
                   (766) 766           780       790       800            816
ATCC35984_Agr      (760) AAAAGATACTTATTGATAATAACAATAGTACTTTTCATATCN-NTATTTTA
Se-2_Agr           (760) AAAAGATACTTATTCATAATAACAATTGTACTTTTTATATCACTTATTTTA
Se-1_Agr           (764) AAAAGATACTTATTCATAATAACAATTGTACTTTTTATATCN-NTATTTTA
Se-3_Agr           (765) AAAAGATACTTATTCATAATAACAATTGTACTTTTTATATCN-NTATTTTA
Consensus          (766) AAAAGATACTTATTCATAATAACAATTGTACTTTTTATATCN NTATTTTA
                                                                  Section 17
                   (817) 817          830       840       850            867
ATCC35984_Agr      (810) CTTTATATGGTGTCACAAACTGATATGCGAGGAAATGATACCCTTAAATTA
Se-2_Agr           (811) CTTTATATGGTGTCACAAACTGATATGCGAGGAAATGATACACTTAAATTA
Se-1_Agr           (814) CTTTATATGGTGTCACAAACTGATATGCGAGGAAATGATACACTTAAATTA
Se-3_Agr           (815) CTTTATATGGTGTCACAAACTGATATGCGAGGAAATGATACACTTAAATTA
Consensus          (817) CTTTATATGGTGTCACAAACTGATATGCGAGGAAATGATACACTTAAATTA
                                                                  Section 18
                   (868) 868         880       890       900            918
ATCC35984_Agr      (861) TATGCCATCTTATTGTTGGGNATTATNNN-TTTTTT---TAGTGTACNGNN
Se-2_Agr           (862) TATGCCATCTNNT-GATGGGNTNNANNNG-TTTTTTAANNNGNNNAGNGAN
Se-1_Agr           (865) TATGCCATCTTGTTGANGGGNNNTTNNNNGTTTTTTTAANNNNTANNGNAT
Se-3_Agr           (866) TATGCCATCTTGTTGANGGNNNTTNNGGN-TTTTTTTANNTNNNNGGNNAT
Consensus          (868) TATGCCATCTTGTTGATGGGNNTTANNNN TTTTTTTANNNNNNNAGNNAT
                                                                  Section 19
                   (919) 919        930       940       950             969
ATCC35984_Agr      (908) AATGANGGATGTCNAATNTACACANNNNGACTTGNGNNNNNNNNNNNNNNN
Se-2_Agr           (911) NTTNAGNGNTGGTNCAATTNNNNNNNNNNNTNNNNNNNNNNNNNNNNNNNN
Se-1_Agr           (916) TNTNGNNGAGNNNNANGTTNNNNNNNNNGNNNNNNNNNNNNNNNNNNNNNN
Se-3_Agr           (916) NNTGNTTGANNGTNNNGTTTNNNNNNNNNNNNNNNNNNNNNNNNNNNNNAN
Consensus          (919) NNTNANNGATNGTNANGTTNNNNNNNNNNNNNNNNNNNNNNNNNNNNNNNN
                                                                  Section 20
                   (970) 970       980       990      1001
ATCC35984_Agr      (959) NNNNNN--------------------------
Se-2_Agr           (962) ANNGNNNAGAAATCNNNNNNNNTNNNANNNNN
Se-1_Agr           (967) NNNNNNNNNNNNNN------------------
Se-3_Agr           (967) NANNNNNNNNNNNNNNNAN-------------
Consensus          (970) NNNNNNNNNNNNNNNNN N
```
