# Supplementary material for: Staphylococcus epidermidis recovered from indwelling catheters exhibit enhanced biofilm dispersal and “self-renewal” through downregulation of agr
Source: BMC Microbiol. 2012 Jun 8;12:102. doi: 10.1186/1471-2180-12-102 (PMC3458918; doi:10.1186/1471-2180-12-102)
Supplement: Additional file 5 — Table S1. Primer sequences for qRT-PCR in this study. [file 1471-2180-12-102-S5.docx]

**Supplemental Table 1. Primer sequences for qRT-PCR in this study.**

| **Gene** | **Sequences (5’ 3’)** |
| --- | --- |
| *RNAIII* | *sense TGAAGTTATGATGGCAGCAGAT*  *antisense GTTGGGATGGCTCAACAACT* |
| *atlE* | *sense AAAGAAACGGCATCTAAT*  *antisense TTCTTCAATTCTTGGTGCTT* |
| *icaA* | *sense AGTTTCAGGCACTAACATCC*  *antisense CGCAGTTACAGGTAATCCAC* |
| *gyrB* | *sense TTATGGTGCTGGACAGATACA*  *antisense CACCGTGAAGACCGCCAGATA* |
| *agr* | *sense GCTGCAACCAAGAAACAACC* |
|  | *antisense CGTGTATTCATAATATGCTTCGATT* |
